# The complete chloroplast genome of *Codonopsis convolvulacea* subsp. *vinciflora*

**DOI:** 10.1080/23802359.2021.1944386

**Published:** 2021-07-01

**Authors:** Yangzom Pema, Chao Ma

**Affiliations:** Tibet Plateau Institute of Biology, Lhasa, Tibet, China

**Keywords:** *Codonopsis convolvulacea* subsp. *vinciflora*, complete chloroplast genome, phylogenetic analysis

## Abstract

*Codonopsis convolvulacea* subsp. *vinciflora* is a commonly used and endangered herb in Tibetan medicine. The chloroplast genome was determined to be 187,634 bp in length and contained a large single-copy and a small single-copy region of 102,174 bp and 8552 bp, respectively. The genome is predicted to contain 140 genes, including 90 protein-coding genes, 42 tRNA genes, and 8 rRNA genes. The overall GC content of the genome is 38.72%. A phylogenetic tree reconstructed by 11 chloroplast genomes reveals that *Codonopsis convolvulacea* subsp. *vinciflora* is mostly related to *C. tsinglingensis* with bootstrap support values of 100%.

*Codonopsis convolvulacea* subsp. *vinciflora* is a perennial herb distributed in the Qinghai-Tibet Plateau and adjacent areas (Tang et al. 2017). As a commonly used bulk herb in Tibetan medical, its main commercial source relies on wild resources, which caused the distribution is severely fragmented and the resource is constantly threatened (Lu and Lan 2013). In this study, the complete chloroplast (cp) genome of this species was sequenced and annotated.

The total genomic DNA was extracted from the fresh leaves of *C. convolvulacea* subsp. *vinciflora* (Lhasa Tibet China, N29°35′27″, E91°6′26″) using the Plant DNA Mini Kit (Genepioneer, Nanjing, China). Specimens were stored in the Herbarium of Tibet Plateau Institute of Biology (Chao Ma, wvoo@163.com) under the accession number: IPBT-20200922MaC35. Total genome DNA was sequenced by a genome sequencing company (Genepioneer Biotechnologies Co. Ltd., Nanjing, China) on the Illumina Hiseq platform. The filtered cp reads were assembled using the program SPAdes assembler 3.10.0 (Bankevich et al. 2012). Annotation was performed using the hmmer v3.1b2 (Eddy 2020), aragorn v1.2.38 (Laslett and Canback 2004), and blast v2.6. using cp genome of *Codonopsis minima* (KY587457) as conference.

The cp genome of *C. convolvulacea* subsp. *vinciflora* was determined to comprise a 187,643 bp double stranded, circular DNA (Accession no. MW336936), contained two inverted repeat (IR) regions of 38,454 bp which were separated by large single-copy (LSC) and small single-copy (SSC) regions of 102,174 bp and 8552 bp, respectively. The cp genome was predicted to contain 140 genes, including 90 protein-coding genes, 42 tRNA genes, and 8 rRNA genes. Among the above predicted coding genes, 11 protein-coding genes, 8 tRNA genes, and 4 rRNA genes were duplicated in IR regions. It was found that 13 genes contained one intron and two genes (clpP and ycf3) contained two introns. The GC content of *C. convolvulacea* subsp. *vinciflora* cp genome was 38.72% in total were 37.7%, 32.78%, and 40.74% for LSC, SSC, and IR regions, respectively.

Ten complete cp genomes of Campanulaceae species were downloaded from NCBI and alignment with *C. convolvulacea* subsp. *vinciflora* using MAFFT (Katoh et al. 2002) online, and a maximum likelihood tree was constructed using FastTree v 2.1.11 (Price et al. 2009, 2010). *C. convolvulacea* subsp. *vinciflora* was found to be mostly related to *C. tsinglingensis* which was highly supported (BS = 100%, Figure 1). The complete cp genome sequence of *C. convolvulacea* subsp. *vinciflora* will provide valuable information for genetic studies of this species.

**Figure 1.**
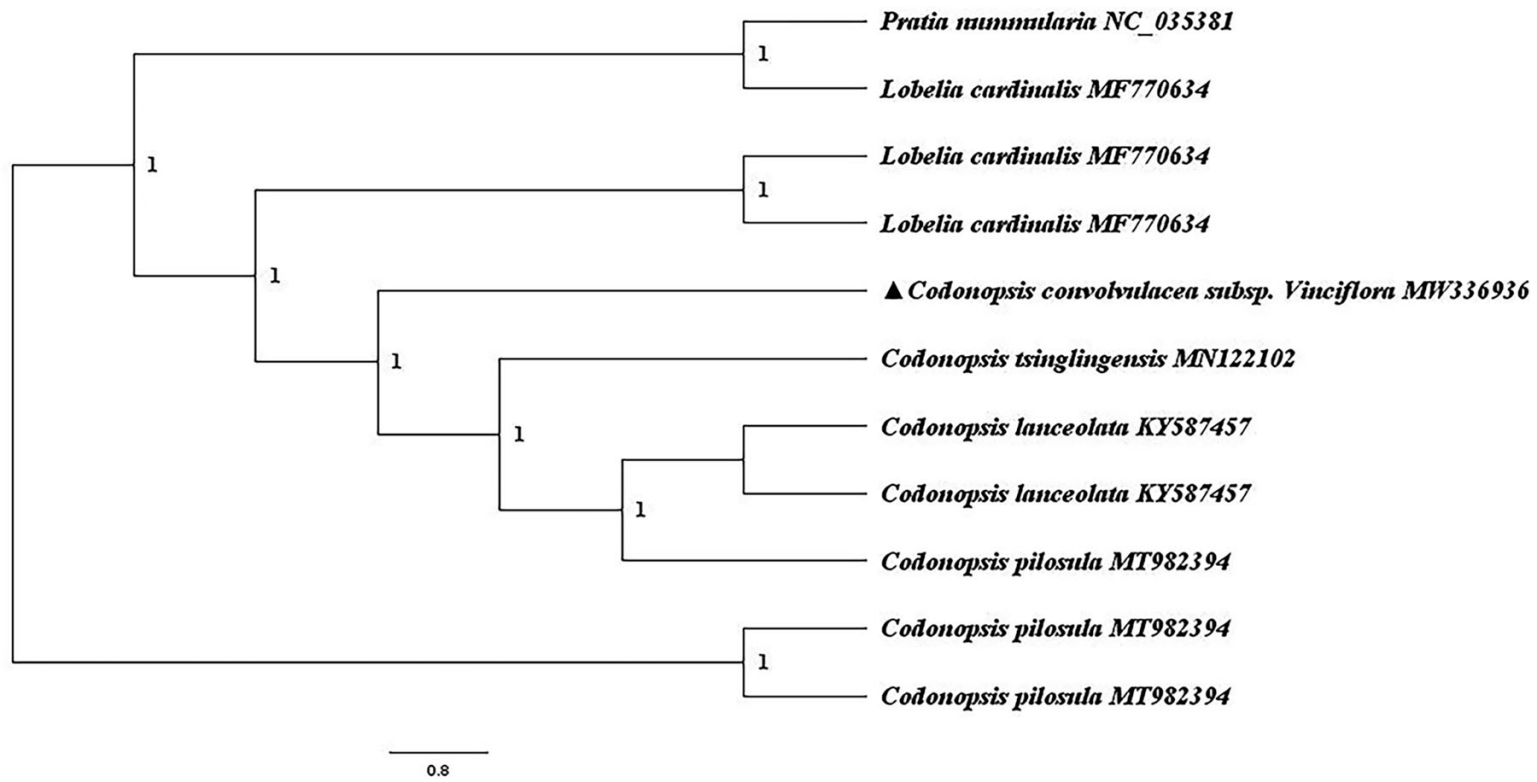
Maximum likelihood (ML) phylogenetic tree based on 11 complete chloroplast genome sequences. The accession numbers showed in the figure, and the triangle indicates that *C. convolvulacea* subsp. *vinciflora* in this study.

## Data Availability

The genome sequence data that support the findings of this study are openly available in GenBank of NCBI at (https://www.ncbi.nlm.nih.gov/nuccore/MW336936.1) under the accession no. MW336936.1. The associated BioProject, SRA, and Bio-Sample numbers are PRJNA722827, SAMN18794340, and SRR14289654, respectively.

## References

[CIT0001] Bankevich A, Nurk S, Antipov D, Gurevich AA, Dvorkin M, Kulikov AS, Lesin VM, Nikolenko SI, Pham S, Prjibelski AD, et al. 2012. SPAdes: a new genome assembly algorithm and its applications to single-cell sequencing. J Comput Biol. 19(5):455–477. 22506599 10.1089/cmb.2012.0021 PMC3342519

[CIT0002] Eddy SR and the HMMER development team. 2020. HMMER: biosequence analysis using profile hidden Markov models. http://hmmer.org/

[CIT0003] Katoh K, Misawa K, Kuma K. i, Miyata T. 2002. MAFFT: a novel method for rapid multiple sequence alignment based on fast Fourier transform. Nucleic Acids Res. 30(14):3059–3066. 12136088 10.1093/nar/gkf436 PMC135756

[CIT0004] Laslett D, Canback B. 2004. ARAGORN, a program to detect tRNA genes and tmRNA genes in nucleotide sequences. Nucleic Acids Res. 32(1):11–16. 14704338 10.1093/nar/gkh152 PMC373265

[CIT0005] Lu J, Lan X. 2013. An investigation on rare and endangered Tibetan medicinal plants in Lhasa region. China J Chin Mater Med. 38(2):127–132. 23596889

[CIT0006] Price MN, Dehal PS, Arkin AP. 2009. FastTree: computing large minimum evolution trees with profiles instead of a distance matrix. Mol Biol Evol. 26(7):1641–1650. 19377059 10.1093/molbev/msp077 PMC2693737

[CIT0007] Price MN, Dehal PS, Arkin AP. 2010. FastTree 2-approximately maximum-likelihood trees for large alignments. PLOS One. 5(3):e9490. 20224823 10.1371/journal.pone.0009490 PMC2835736

[CIT0008] Tang X, Lu J, Li L, Lan X. 2017. Resource characteristics and sustainable utilization of *Codonopsis convolve*, a Tibetan medicinal plant. Agric Sci Technol. 18(2):1382–1390.

